# Neural encoding of saltatory pneumotactile velocity in human glabrous hand

**DOI:** 10.1371/journal.pone.0183532

**Published:** 2017-08-25

**Authors:** Hyuntaek Oh, Rebecca Custead, Yingying Wang, Steven Barlow

**Affiliations:** 1 Department of Biological Systems Engineering, University of Nebraska, Lincoln, Nebraska, United States of America; 2 Department of Special Education and Communication Disorders, University of Nebraska, Lincoln, Nebraska, United States of America; 3 Center for Brain, Biology and Behavior, University of Nebraska, Lincoln, Nebraska, United States of America; Boston Children’s Hospital / Harvard Medical School, UNITED STATES

## Abstract

Neurons in the somatosensory cortex are exquisitely sensitive to mechanical stimulation of the skin surface. The location, velocity, direction, and adaptation of tactile stimuli on the skin’s surface are discriminable features of somatosensory processing, however the representation and processing of dynamic tactile arrays in the human somatosensory cortex are poorly understood. The principal aim of this study was to map the relation between dynamic saltatory pneumatic stimuli at discrete traverse velocities on the glabrous hand and the resultant pattern of evoked BOLD response in the human brain. Moreover, we hypothesized that the hand representation in contralateral Brodmann Area (BA) 3b would show a significant dependence on stimulus velocity. Saltatory pneumatic pulses (60 ms duration, 9.5 ms rise/fall) were repetitively sequenced through a 7-channel TAC-Cell array at traverse velocities of 5, 25, and 65 cm/s on the glabrous hand initiated at the tips of D2 (index finger) and D3 (middle finger) and sequenced towards the D1 (thumb). The resulting hemodynamic response was sampled during 3 functional MRI scans (BOLD) in 20 neurotypical right-handed adults at 3T. Results from each subject were inserted to the one-way ANOVA within-subjects and one sample t-test to evaluate the group main effect of all three velocities stimuli and each of three different velocities, respectively. The stimulus evoked BOLD response revealed a dynamic representation of saltatory pneumotactile stimulus velocity in a network consisting of the contralateral primary hand somatosensory cortex (BA3b), associated primary motor cortex (BA4), posterior insula, and ipsilateral deep cerebellum. The spatial extent of this network was greatest at the 5 and 25 cm/s pneumotactile stimulus velocities.

## Introduction

Animal and human models of brain plasticity have shown that the development of functional motor tasks depend on the interplay between sensory input and motor output [1, 2]. Among the many functions of the somatosensory system, processing information about the location, velocity and traverse length of tactile stimuli on the body surface is presumed essential for the development and maintenance of fine motor control of the hand [3–5]. Improving our knowledge of velocity and directional encoding in this sensory domain will help formulate innovative neurotherapeutic strategies for the rehabilitation of brain-damaged patients to regain motor skills in the limb (hand, foot) and orofacial (speech, gesture, swallowing) systems [6]. Limited data exist on the cortical representation of moving touch stimulation on the glabrous skin of the digits in humans [7, 8], and many studies involving sensorimotor tasks have been limited to neurotypical adults using electrical and/or transcranial magnetic stimulation (TMS) [9–11].

The sensory flow of tactile information derived from mechanoreceptors in the glabrous skin of the hand is conveyed along the dorsal column-medial lemniscus and transmitted through the contralateral ventroposterolateral (VPL) thalamus and primary somatosensory cortex (S1), whereas the secondary somatosensory cortex (S2) typically shows a bilateral response to a unilateral somatosensory stimulus [12, 13]. Many neurons in the posterior parietal cortex (PPC) respond to both tactile and visual inputs [14, 15], with select sensorimotor transformation and output to the premotor cortex (PMC) [16]. The cerebellum represents the ‘forward model’ of the sensorimotor system that implements predictions of the sensory result from the motor commands, and these predictions can be used to improve a motor skill or activate sensorimotor plasticity [17, 18]. Several neuroimaging studies using functional Magnetic Resonance Imaging (fMRI) and positron emission tomography (PET) have discovered that the cerebellum is involved in signaling the sensory consequence of movements resulting from the correlation between the actual and predicted sensory feedback, and forward models stored in the cerebellum are related to predictions of movements [19, 20]. Since the cerebellum plays an important role in predictive motor control and storing forward models [21, 22], recent human studies highlight the crucial role of the cerebellum and sensorimotor cortex during motor learning and functional recovery from stroke [23, 24].

Moving tactile stimulation on glabrous skin, known historically as ‘surface parallel stimulation’ [4], has been shown to evoke activity among cortical and subcortical somatosensory representations [25]. Human psychophysical studies have shown that the optimal range of stimulus velocity for the discrimination of skin traverse velocity lies between 3 and 25 cm/s [3, 4, 26, 27]. Similar velocities of brush stroke stimuli have been used to map the Blood-oxygen-level dependent (BOLD) responses in S1 and posterior insular cortex [28, 29]. Beyond this optimal range, neurotypical subjects were still able to recognize the brushing stimuli at velocities exceeding 50 cm/s, however the perception of discrimination of velocity became less reliable due to changes in perceived stimulation location, direction, and distance. At low velocities (e.g., < 3 cm/s), S1 neurons appear to encode the moving tactile stimulation as discrete stimulus events rather than a progressive traverse motion track. Furthermore, an accurate discrimination of skin velocity on glabrous skin of the hand may yield better encoding over a wider range of velocities compared to the hairy skin since A*β* mechanoreceptors in the glabrous skin are superior at encoding the temporal and spatial properties of incoming stimuli [27, 30, 31]. Thus, a consideration of the optimal operating range for velocity and direction of moving tactile stimulation on the glabrous hand are important factors to consider when designing a perceptual or functional imaging experiment with human subjects [32, 33].

The glabrous hand and orofacial skin feature high innervation densities, large number of receptive fields, and acute sensitivity which translate to high cortical magnification in S1 [34]. Many neuroimaging modalities such as 1.5 T fMRI, magnetoencephalography (MEG), or PET of the human brain do not provide enough spatial resolution to map individual fingers and their phalanges because the distances between individual digits and segments represented in S1 are only a few mm [35]. Thus, high resolution 3T fMRI with multichannel head coils are better equipped to achieve small voxel size combined with precisely controlled dynamic spatial tactile arrays to map the hand-finger somatotopy under conditions where velocity and/or direction are independent variables of interest [36, 37]. There are inherent challenges in the design of an MRI-compatible tactile stimulus array control system that is scalable for velocity and direction. A limited number of studies have explored tactile encoding using continuous moving brush, piezo-element vibration, and compressed air [38–42]. Thus, in order to advance our understanding of tactile velocity encoding networks in the human brain, the need exists for a programmable, multichannel tactile stimulus control system that is non-invasive, simple to configure and can be applied anywhere on the body with scalable velocity control and fully MRI compatibility.

The primary goal of the present study was to functionally map the human brain to identify the relation between saltatory pneumotactile stimulation at 3 velocities on the glabrous hand and the evoked hemodynamic BOLD response in select regions of interest (ROIs), including cerebral somatosensory areas (S1, S2, PPC, posterior insula), and deep cerebellum among 20 neurotypical adults using high-resolution fMRI methods. In this study, three velocities, including a relatively low but not discrete velocity (5cm/s), a medium velocity (25cm/s), and a relatively high but perceptable velocity (65cm/s), were chosen to investigate the dynamic BOLD response between low and high end of perceivable velocity range. We hypothesized that the somatosensory network would show evidence of modulation as reflected in %BOLD change among the ROIs of interest as a function of saltatory velocity. To achieve this objective, a 7-channel TAC-Cell array developed in our laboratory was configured to the glabrous hand on three digits, including D1 (thumb), D2 (index finger), and D3 (middle finger) for saltatory pneumatic stimulation randomized at 3 velocities.

## Materials and methods

### Subjects

Twenty right-handed, neurotypical adults (14 females, 6 males) age 18-30 years (mean = 22.3±2.47 years) participated in this study. Exclusion criteria: traumatic injury to the hand or neurological disease resulting in sensorimotor impairment affecting hand movement and/or sensory function. Each subject provided informed written consent in accordance with the University of Nebraska—Lincoln institutional review board approval.

### Stimulus device: Galileo somatosensory pneumatic stimulus control system

**Fig 1:**
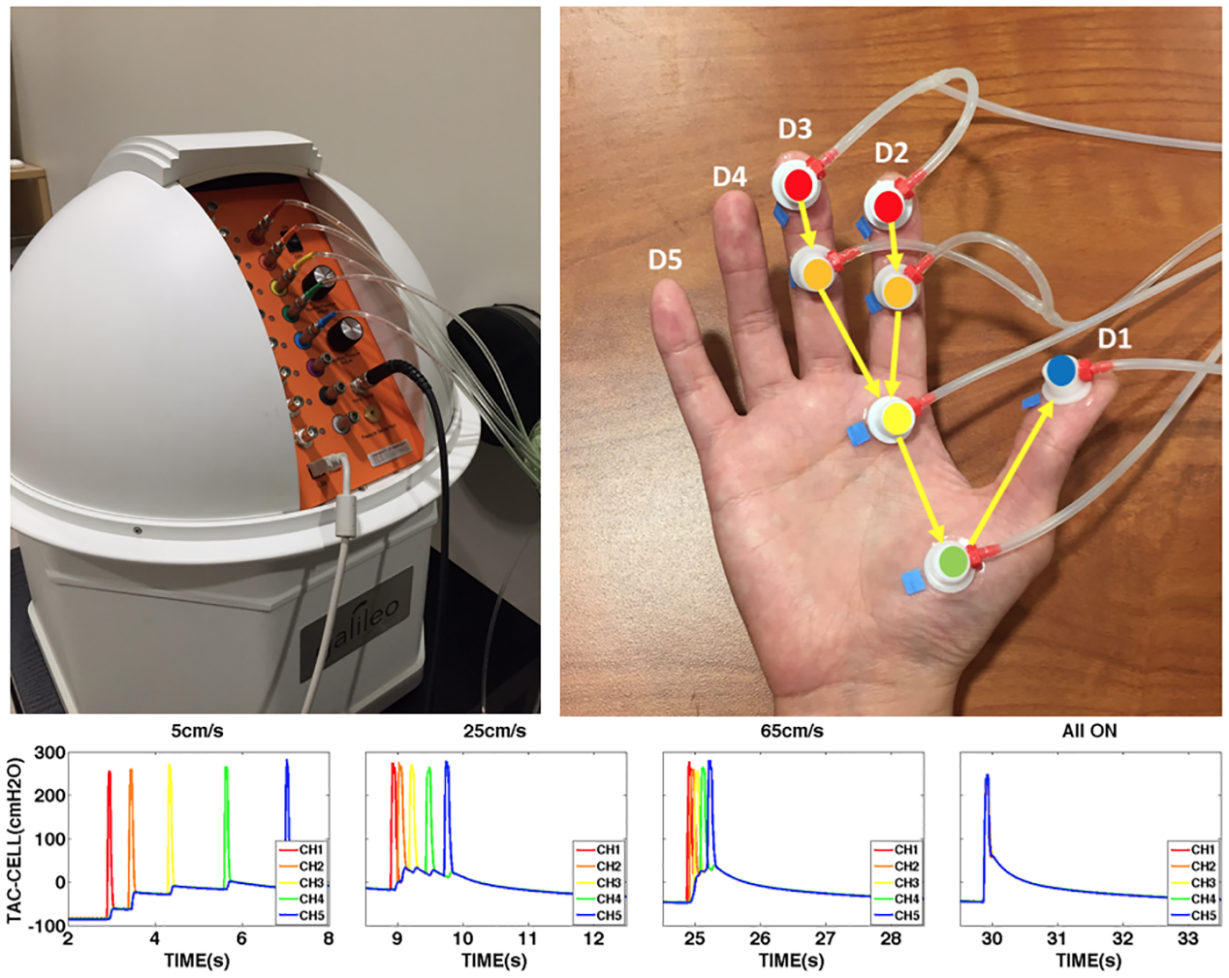
Galileo somatosensory^™^ tactile stimulation. Top Left: Galileo somatosensory^™^ tactile stimulator. Top Right: Stimulated areas and TAC-Cells location. p1 in D2 and D3 = red, p2 in D2 and D3 = orange, p4 in D2 and D3 = yellow, p4 in D1 = green, p1 in D1 = blue. The TAC-Cell is essentially a small capsule (OD = 15 mm, ID = 6 mm), and machined from acetal thermoplastic. Bottom: Stimulus velocity pressure waveforms for each condition.

A multichannel pneumatic amplifier and tactile array known as the Galileo Somatosensory^™^ system (Epic Medical Concepts & Innovations, Inc., Mission, Kansas USA) was used for mechanosensory stimulus generation. The Galileo features scalable pulse generation in configurable arrays, and is fully MRI/MEG compatible. The pneumatic stimulator probes, known as TAC-Cells, are made from acetyl thermoplastic homopolymer, use tiny volumes of compressed air to rapidly deform (10 ms rise/fall times) the surface of the skin. The individual pressure pulses generated by the Galileo controller are transmitted through 18’ of polyurethane tubing (3/32” ID) which is routed through a waveguide into the MRI suite and terminated with TAC-Cells to allow for placement on the subject’s hand with the bore of the MRI scanner. The PC laptop computer, Galileo SomatosensoryTM pneumatic controller, and integrated dual-cylinder pump motor are all located outside the shielded MRI scanner suite room. As shown in Fig 1, the TAC-Cell is essentially a small capsule with a sealing flange (6 mm ID, 15 mm OD), which can be adhered rapidly to virtually any skin surface, including the glabrous hand and face [6, 43, 44].

### fMRI data acquisition

A brain structural MRI scan and 3 functional sessions (BOLD) were recorded at 3.0 T (Skyra, Siemens Medical Solutions, Erlangen, Germany) using a 32-channel head coil. Structural T1-weighted 3-dimensional image of the subject’s brain (MPRAGE, Magnetization-Prepared Rapid Gradient-Echo) was acquired at the beginning of the session [repetition time (TR) = 2400 ms, echo time (TE) = 3.37 ms, voxel size = 1 x 1 x 1 mm, flip angle = 7°, number of slices = 192, acquisition matrix = 256 x 256, field of view (FoV) = 256 x 256 mm, total acquisition time (TA) = 5:35 minutes].

Following the MPRAGE anatomical scan, three sessions of functional images were recorded using a T2*-weighted EPI (Echo Planar Imaging) sequence [TR = 2500 ms, TE = 30 ms, voxel size = 2.5 x 2.5 x 2.5 mm, flip angle = 83°, number of slices = 320, acquisition matrix = 88 x 88, FoV = 220 x 220 mm, Phase partial Fourier factor = 7/8, TA = 13:53].

Visual countdown presentation to maintain the subjects’ vigilance was performed using E-prime 2.0 software (Psychology Software Tools, Inc., Sharpsburg, PA, USA). This visual presentation was projected onto a screen behind (headward) the scanner bore. The subject observed the presentation on a mirror which was attached to the 32-channel head coil. The visual countdown presentation included a declining sequence of numbers (20:1) which corresponds to the number of remaining stimulus blocks in the BOLD session. The number on the presentation was shown only for 0.5 second to minimize a primary visual cortex response.

### Tactile stimulus control

Seven small plastic pneumatic TAC-Cells (6mm ID) were placed on the palm of the right hand along the length of index and middle finger using tincture of Benzoin (10% concentration to increase adhesion) followed by the application of double adhesive tape collars. A Galileo^™^ Somatosensory tactile array was programmed to deliver punctate (60 ms duration, 9 ms rise/fall) pneumotactile sequence through TAC-Cells placed on the glabrous skin of the right hand (see Fig 1), including p1, p2 segments of D3 (middle finger), p1, p2, p4 segments of D2 (index finger), and p4, p1 of D1 (thumb). Morphometric dimensions between p1 and p2 in D2 (Length 1), p2 and p3 in D2 (Length 2), p4 in D2 and P4 in D1 (Length 3), and p4 and p1 in D1 (Length 4) were measured from each subject to adjust for variations in hand size to create accurate tactile traverse velocities (Fig 1). Programmed time delays between individual TAC-Cells result in a saltatory velocity sequence traversing the tips of D1, D2 through the basal phalangeal segments to the distal phalanx of the thumb. The silicon tubing was bifurcated at its terminal for channels 1 and 2 to deliver a pneumotactile stimulus on the p1 and p2 segments of the D2 and D3. Rice-filled hand-warmers placed within mitten gloves were fit to all subject’s right hand to maintain normothermia of limb extremities during testing in the MRI scanner suite [45]. It is through this array of pneumatically charged TAC-Cells that the subject experienced repeated trains of saltatory pulsed pneumotactile stimulation ranging from very slow (5 cm/s) to fast (65 cm/s) traverse speeds on the glabrous surface of the hand.

**Fig 2:**
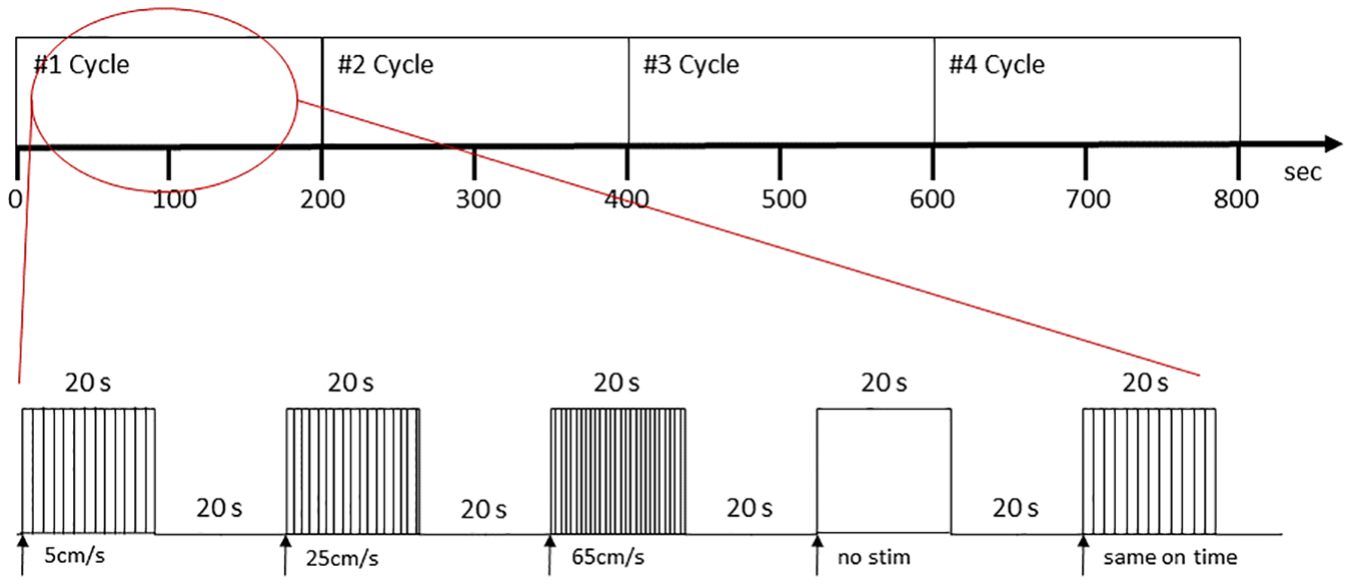
Experiment stimulus design. Random-balanced experimental block design in one functional scan session. One session includes 4 cycles of the 5 stimulus conditions and the total measurement time of one session was 13:20 min.

A randomized-balanced block design (40 sec duration/block) included the following 5 conditions: Saltatory velocities @ 5, 25, and 65 cm/sec, simultaneous TAC-Cells ON, and all cells OFF (Fig 2). There were three sessions during the fMRI BOLD response acquisition and each session included 4 cycles of the 5 stimulus conditions. Thus, a total of 20 conditions in each session were counter-balanced and randomized. The duration of the stimulus event for each condition was 20 seconds (8 volumes, TR = 2500 ms), followed by 20 seconds of rest. The tactile stimulus was continuously delivered from p1 of D2 and D3 to p1 of D1, passing through both p2 of D2 and D3, p4 of D2 and D3, p4 of D2 and p4 of D1. The average time elapsed between velocity trains of 5, 25 and 65 cm/sec were 501.1 ms, 153 ms, and 37.6 ms, respectively. Total BOLD sampling time of one session was 13:20 min (320 volumes), thus 3 BOLD acquisitions produced 960 volumes of fMRI data per subject.

### fMRI data analysis

Pre-processing and statistical analysis of MPRAGE and functional images were performed using SPM 12 (Statistical Parametric Mapping; Wellcome Department of Imaging Neuroscience, London, UK). The 3 sessions of functional MRI volumes were realigned to the first volume in the each session, normalized to adjust overall size and orientation of the functional and anatomical images to the MNI template, and smoothed by convolution with an isotropic Gaussian kernel (FWHM = 8 mm).

The General Linear Model (GLM) was applied to estimate the predictor variables by convolving the design matrix (the box-car stimulus blocks) and the hemodynamic response function for the single-subject analysis of BOLD responses from different velocities of tactile stimulus [46]. The model includes five regressors (5 cm/s, 25 cm/s, 65 cm/s, all TAC-Cells OFF, and all TAC-Cells ON), and six motion parameter correction regressors (three translational axes [X, Y, Z] and three rotations [roll, yaw, pitch]) per session. One-sided main effect for each velocity condition was determined by subtracting the no stimulus block contrast (control block). Resulting t-maps from each BOLD session were carried forward to the Mixed Effects (MFX) analysis to combine the 3 BOLD results within a subject [47]. An F-contrast was required to determine the main effect of velocity conditions. The result from F-contrasts showed how the different stimulus velocities change brain response and where the stimulus influences the BOLD response in the brain. Besides the result from F-contrasts, six additional contrasts were created: 1) 5 cm/s > No stimulus, 2) 25 cm/s > No stimulus, 3) 65 cm/s > No stimulus, 4) 5 cm/s > All TAC-Cells ON, 5) 25 cm/s > All TAC-Cells ON, and 6) 65 cm/s > All TAC-Cells ON. The contrast results from each subject were entered into the 2nd-level analysis to access the group analysis. On SPM group analysis, an uncorrected p-value = .0001 was used. The group analysis of one-sided main effects for 5 cm/s, 25 cm/s and 65 cm/s accepted the one sample t-test which was used to compute within-subject contrast results from 1st-level analysis. One-way ANOVA analysis was implemented to derive the group main effect among the various velocity stimulus profiles. The t-contrast results from each subject were used in the one-way ANOVA analysis.

## Results

The seven TAC-Cells, configured to digits D1, D2, and D3 of the glabrous right hand which were programmed to produce 3 saltatory velocities (5, 25, 65 cm/s) were highly effective in evoking a scalable BOLD response among several ROIs within the human somatosensory network.

The first-level result from each single subject was acquired by combining 3 BOLD sessions with the exception of one subject who had 2 BOLD sessions. The significant level was set to P_unc_ <.0001 for the five stimulus conditions (5 cm/s, 25 cm/s, 65 cm/s, All-Off (No stimulus), and All-ON). A dominant contralateral response among the velocity conditions was consistently found in the majority of single subject BOLD activations (19/20 subjects). Significant BOLD responses were localized to the sensorimotor cortex which includes the postcentral gyrus (S1, S2), primary and premotor cortex, posterior insula, and deep cerebellum. For the 25 cm/s stimulus condition, BOLD responses were found in the insula in 13/20 subjects. The spatial extent of the evoked BOLD response was significantly dependent on saltatory tactile velocity with the largest response apparent at 25 cm/s. The probabilistic cytoarchitectonic maps in SPM Anatomy toolbox v2.2b were used to identify the brain region corresponding to the peak MNI coordinates from the main effects results [48–51].

### Main effect of various velocity stimuli

**Fig 3:**
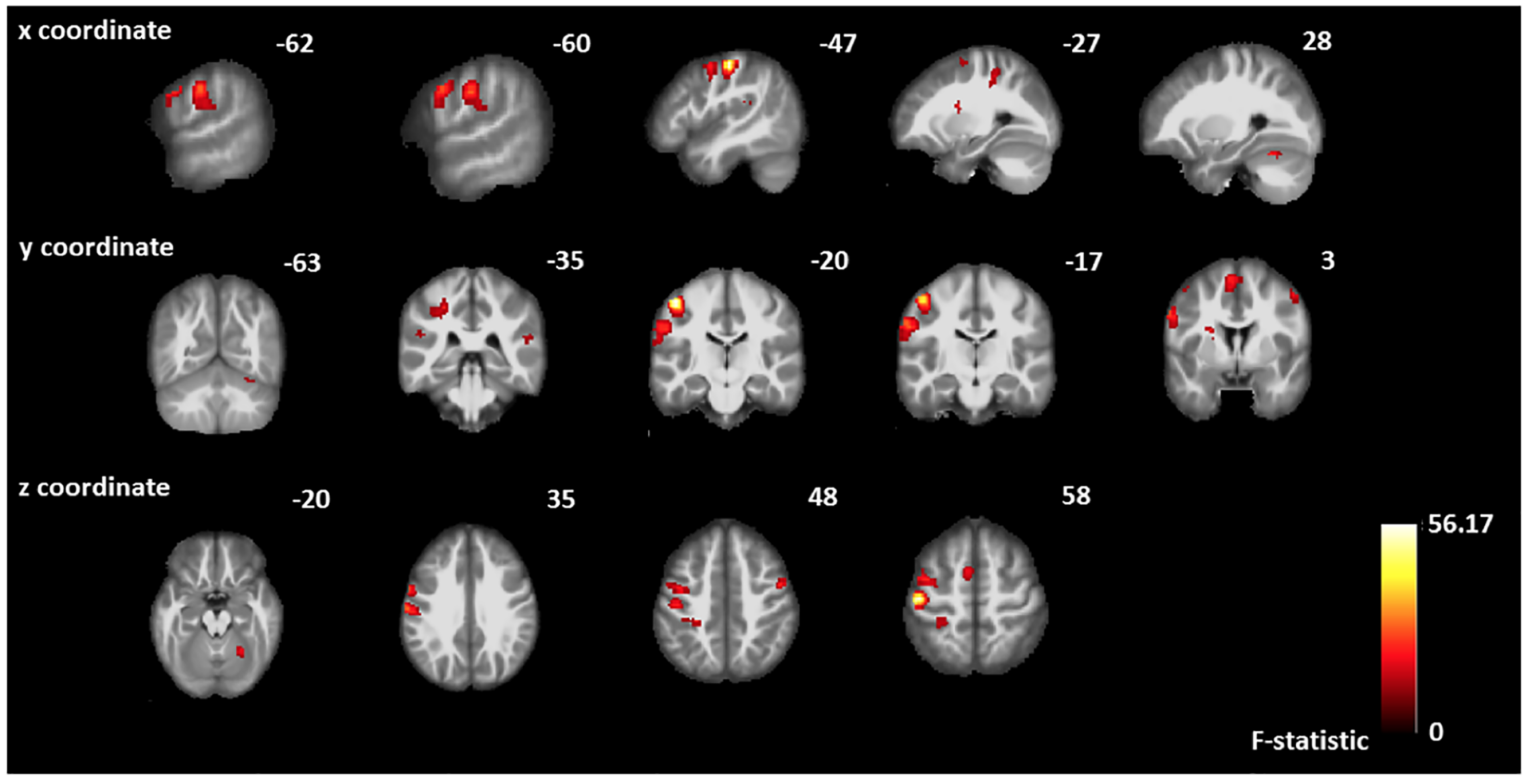
The main effect of velocity stimuli. The main effect of velocity from 20 neurotypical subjects combining 3 different velocities stimulus (5 cm/s, 25 cm/s, and 65 cm/s). Color-coded evoked BOLD responses at each row indicate brain regions (sagittal, coronal and axial view) with high F-values. Most of the BOLD responses in Table 1 are represented in this figure.

The t-contrast results from main effects for 5 cm/s, 25 cm/s, and 65 cm/s were inserted in a one-way ANOVA within-subjects analysis to evaluate the group main effect of various velocity stimuli with significance level set to P_unc_ <.0001. The result of the group main effect was used to identify responsive S1, S2 and the somatosensory association areas. Fig 3 shows the BOLD response of the main effect of velocity in both cortical activation and its coronal view. The MNI coordinates and F-values of the main effect of the velocity are listed in Table 1. The result from the one-way ANOVA within-subjects showed BOLD responses in not only contralateral and ipsilateral cerebral sensorimotor area (S1, S2, primary motor cortex (M1), supplementary motor cortex (SMA), posterior insula and postcentral gyrus), but also ipsilateral cerebellum. The peak level of contralateral BOLD response was found in BA3b [MNI (mm) = -47, -20, 58; F = 56.18], followed by postcentral gyrus [MNI (mm) = -62, -17, 35; F = 28.21]. The highest level of ipsilateral BOLD response was found in the precentral gyrus [MNI (mm) = 51, 1, 50; F = 28.99] followed by cerebellum near the dentate nucleus [MNI (mm) = 26, -55, -23; F = 26.97].

**Table 1:** Main effect of the velocity MNI coordinates.

MNI Coordinates			Cluster-level		Peak-level			Region
x	y	z	P_FWE-corr_	Extent (k_E_)	F-value	Z	P_uncorr_	
-47	-20	58	0.000	163	56.18	6.82	0.000	L BA3b
51	1	50	0.005	56	28.99	5.47	0.000	R Precentral Gyrus
-62	-17	35	0.000	193	28.21	5.41	0.000	L BA1
26	-55	-23	0.016	39	26.97	5.32	0.000	R Cerebellum
-60	3	35	0.000	293	26.78	5.31	0.000	L BA44
-45	-5	53			26.11	5.26	0.000	L Precentral Gyrus
-55	-2	43			15.61	4.73	0.000	L Precentral Gyrus
-5	1	65	0.000	130	25.01	5.17	0.000	L BA6
-27	-35	48	0.000	106	23.29	5.03	0.000	L BA3a
-35	-35	43			15.61	4.24	0.000	L BA3a
-30	-40	58			15.46	4.22	0.000	L BA2
-50	-37	23	0.056	24	15.40	4.21	0.000	L Superior Temporal Gyrus
56	-35	20	0.097	18	14.99	4.16	0.000	R Superior Temporal Gyrus

One-way ANOVA within-subjects revealed a significant (p (peak-level) <.0001, uncorrected) main effect of the saltatory pneumotactile velocity stimulation. Cluster-level: The number of activated voxels comprising a cluster. Peak-level: The height of maximum voxel within the cluster, P_FWE-corr_: family-wise error correction, Extent threshold k > 10 voxels, P_uncorr_: uncorrected, BA: Brodmann area, L: Left, R: Right.

### BOLD signal changes and time series

**Fig 4:**
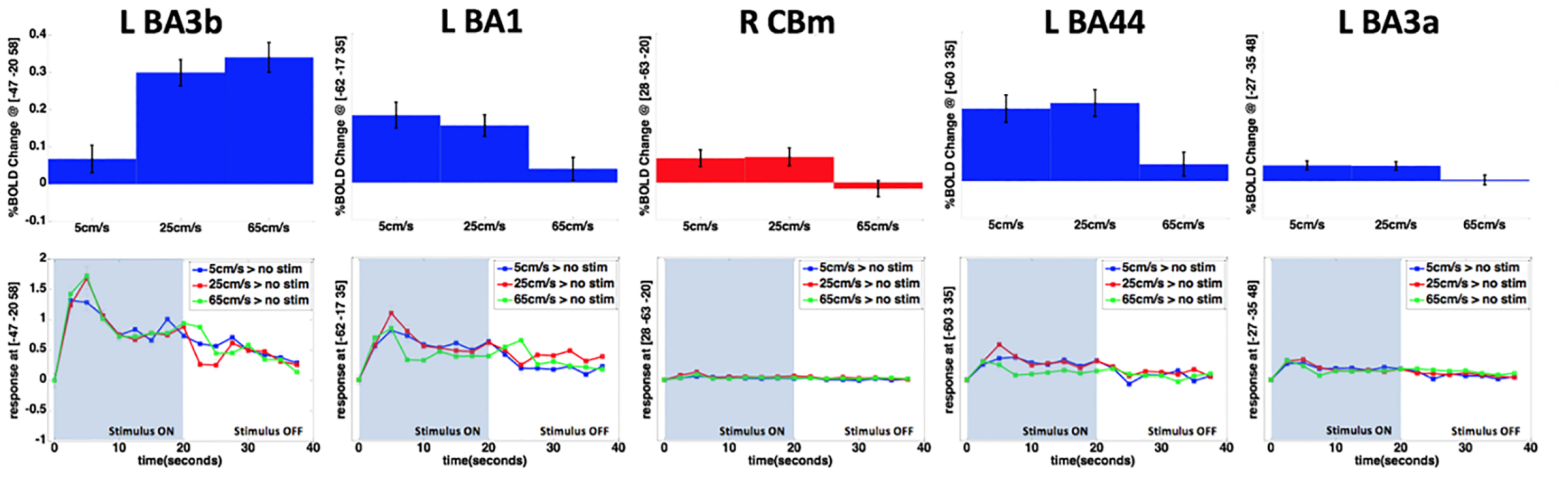
BOLD signal changes and BOLD response time courses. Top: The bar graphs show the BOLD signal changes of 3 velocities compared to rest (no stimulus) in Left BA3b, Left BA1, Right Cerebellum, Left BA44 and Left BA3a with SEM (estimated as the mean of percentage BOLD signal changes across the 20 seconds stimulus block, P_unc_ <.0001. Blue and Red indicate contralateral and ipsilateral to the stimulus, respectively). Bottom: BOLD response time courses corresponding with each area from the BOLD signal changes (estimated as the average BOLD responses across 20 subjects during the 40 seconds block including stimulus ON and OFF, Blue = 5 cm/s > NO stimulus, Red = 25 cm/s > No stimulus, Green = 65 cm/s > No stimulus). Y-scales are same for five figures in each row (% BOLD change and BOLD response time courses).

The peak MNI coordinates of left BA3b (-47, -20, 58), BA1 (-62, -17, 35), BA44 (-60, 3, 35), BA3a (-27, -35, 48), and right Cerebellum (26, -55, -23) were selected from the result of the main effect for stimulus velocity. The resulting MNI coordinates were estimated with 34%, 39%, 12%, 27% and 98% probability for the left BA3b, left BA1, left BA44, left BA3a, and right cerebellum, respectively, by using the ANATOMY toolbox v2.2b. Fig 4 shows the BOLD signal changes and BOLD response time courses among the 20 subjects pooled for each of the 3 conditions compared to rest (no stimulus) in left BA3b, BA1, BA44, BA3a, and right cerebellum (estimated as the mean of percentage BOLD signal changes across the 20 seconds stimulus block, P_unc_ <.0001). The %BOLD signal changes for each area were calculated by using the ANATOMY toolbox v2.2b. The largest %BOLD signal changes as a function of saltatory pneumotactile velocity were found at left BA3b, followed by left BA44 and BA1. The smallest %BOLD signal changes in left BA3b were found for the 5 cm/s and no stimulation contrast. The %BOLD signal change in the left BA3b increased as a function of saltation velocity. The pattern of BOLD modulation in the left BA1, however, was reversed with greatest BOLD signal change associated with the 5 cm/s contrast and progressively smaller BOLD signal change at 25 cm/s and 65 cm/s. The right deep cerebellum, left BA44, and left BA3a showed significant BOLD signals at the 5 and 25 cm/sec saltation rates, with attenuation of the evoked BOLD response at the highest velocity of 65 cm/s. The peak BOLD response in these five ROI time series were found 5 seconds after stimulus onset with the ‘65 cm/s > No stimulus’ contrast showing the greatest BOLD response in left BA3b. Most of the %BOLD signal change results are generally consistent with the BOLD time series functions (Fig 4).

### One sample t-test (velocities > no stimulus)

**Fig 5:**
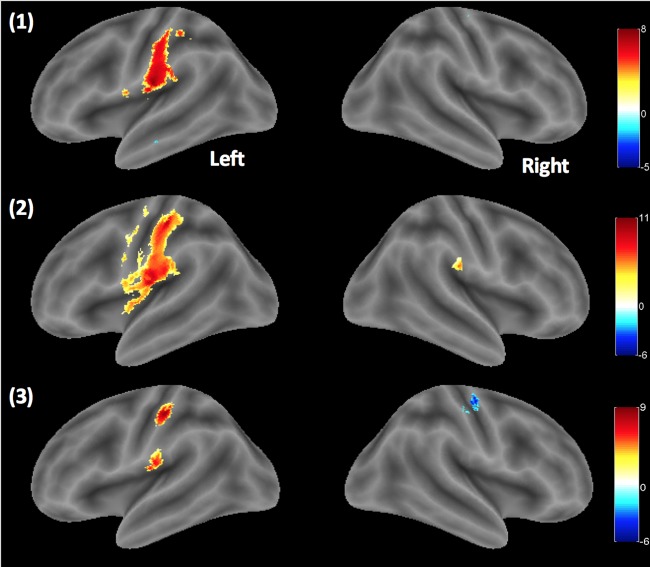
Group results. Velocities > no stimulus. One sample t-test result on the rendered brain cortical surface using bspmview (http://www.bobspunt.com/bspmview/) [from the top: (1) 5 cm/s, (2) 25 cm/s, and (3) 65 cm/s > No stimulus, P_unc_ <.0001].

The results from one sample t-test in the second-level analysis showed a group result of one-sided individual velocities compared to the two control conditions (All TAC-Cell pneumatics OFF and ON). When the individual velocities were compared to the All TAC-Cells OFF condition (No stimulus) in Fig 5 (the contrasts: 5 cm/s > All TAC-Cells OFF, 25 cm/s > All TAC-Cells OFF, and 65 cm/s > All TAC-Cells OFF), the contralateral BOLD activations in sensorimotor cortex were found consistently across most subjects, with the largest spatial extent and t-values of the evoked BOLD responses at ‘25 cm/s > All TAC-Cells OFF’. MNI coordinates, t-value, and brain regions are listed in Table 2. Contralateral BOLD responses localized predominantly to sensorimotor cortex (BA1, BA2, and pre- and postcentral gyrus) were found in both ‘5 cm/s > No stimulus’ and ‘65 cm/s > No stimulus’ contrasts, whereas ‘25 cm/s > No stimulus’ contrast evoked significant BOLD responses in BA1, BA43 (a portion of S2 proximal to the posterior end of the lateral fissure of Sylvius) and postcentral gyrus. The ipsilateral BOLD responses were found in the inferior parietal lobule (IPL) only at ‘25 cm/s > No stimulus’ [MNI (mm) = 53, -27, 23; t = 8.22].

**Table 2:** One sample t-test results. Velocities > no stimulus.

	MNI Coordinates			t-value	P_uncorr_	Region
	x	y	z			
**5cm/s > No stimulus**	-65	-20	43	8.70	0.000	L BA1
	-52	-17	20	8.64	0.000	L Postcentral Gyrus
	-57	-22	50	6.80	0.000	L BA1
	-37	-35	45	5.89	0.000	L BA2
	-50	-5	8	5.66	0.000	L Precentral Gyrus
	-45	-5	15	5.04	0.000	L Rolandic Operculum
**25cm/s > No stimulus**	-50	-25	55	11.92	0.000	L BA1
	-47	-17	18	10.83	0.000	L BA43
	-55	-15	20	10.31	0.000	L Postcentral Gyrus
	53	-27	23	8.22	0.000	R Inferior Parietal Lobule
**65cm/s > No stimulus**	-52	-22	55	9.40	0.000	L BA1
	-47	-17	20	6.98	0.000	L Rolandic Operculum (OP3)

One sample t-test revealed a significant (p<0.0001, uncorrected) BOLD response of one-sided individual velocities compared to all TAC-Cells OFF (No stimulus). BA: Brodmann area, L: Left, R: Right, P_uncorr_: uncorrected.

### One sample t-test (velocities > all on)

**Fig 6:**
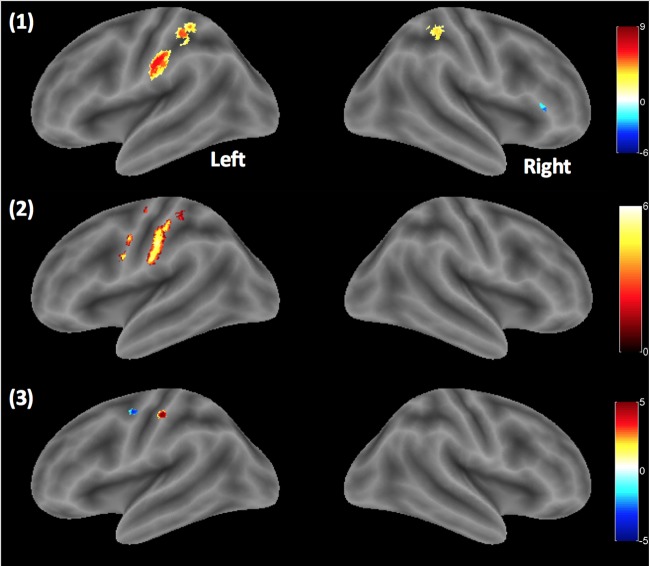
Group results. Velocities > all-ON. One sample t-test result on the rendered brain cortical surface using bspmview (http://www.bobspunt.com/bspmview/) [from the top: (1) 5 cm/s, (2) 25 cm/s, and (3) 65 cm/s > All-ON, P_unc_ <.0001].

The individual velocities were compared to the All TAC-Cells ON condition as shown in Fig 6. The contralateral BOLD responses in sensorimotor cortex (BA3, BA6 and pre- and postcentral gyrus) were found for the three contrasts: ‘5 cm/s > All TAC-Cells ON’, ‘25 cm/s > All TAC-Cells ON’, and ‘65 cm/s > All TAC-Cells ON’, whereas the ipsilateral BOLD activations in BA2 were seen only at ‘5 cm/s > All TAC-Cells ON’ [MNI (mm) = 33, -37, 45; t = 6.35]. As shown in Table 3, the peak t-value was observed at ‘5 cm/s > All TAC-Cells ON’ (t = 9.18) while relatively small BOLD responses were found at the highest velocity condition (65 cm/s > All TAC-Cells ON). The spatial extent of BOLD responses at ‘5 cm/s > All TAC-Cells ON’ and ‘25 cm/s > All TAC-Cells ON’ were larger than the highest velocity contrast.

**Table 3:** One sample t-test results. Velocities > all-ON.

	MNI Coordinates			t-value	P_uncorr_	Region
	x	y	z			
**5cm/s > All-ON**	-67	-17	33	9.18	0.000	L Superior Temporal Gyrus
	33	-37	45	6.35	0.000	R BA2
	-40	-35	48	5.68	0.000	L BA2
	-32	-32	45	5.57	0.000	L BA3a
	-30	-10	58	4.99	0.000	L BA6
	-25	-7	50	4.71	0.000	L BA6
**25cm/s > All-ON**	-60	-17	40	6.96	0.000	L BA1
	-57	-2	45	6.66	0.000	L BA6
	-62	-15	30	6.23	0.000	L BA1
	-37	-10	65	5.26	0.000	L Precentral Gyrus
**65cm/s > All-ON**	-47	-20	58	5.75	0.000	L BA3b

One sample t-test revealed a significant (p<0.0001, uncorrected) BOLD response of one-sided individual velocities compared to all TAC-Cells ON. BA: Brodmann area, L: Left, R: Right, P_uncorr_: uncorrected.

## Discussion

In this study, we used a new saltatory pneumotactile stimulus modality programmed at 3 different velocities on the glabrous hand to map the evoked hemodynamic BOLD response in cortical and subcortical somatosensory areas using fMRI methods. Overall, the BOLD main effect for saltatory pneumotactile velocity was localized to several loci involving contralateral and bilateral cerebral cortex, and ipsilateral cerebellum. This elaborate network extends previous observations based on fMRI and MEG of pneumotactile encoding of single channel pulse train inputs (non-saltatory) which found principal dipoles localized to S1, S2 and PPC in both contralateral and bilateral cerebral sensorimotor cortex [52, 53]. Additionally, the cerebral responses in S1 and PPC are generally consistent with the findings from our previous MEG studies using the first and second generation of TAC-Cells (19.3 mm ID, and 6 mm ID, respectively) developed in our laboratory [43, 44, 53]. We also found significant ipsilateral BOLD responses in deep cerebellum which was reported in previous fMRI and PET studies using the brush and the foam-tipped motor to create the movement of the tactile stimulus (tickling) on the palm [17, 19, 54]. Moreover, our pneumotactile saltatory stimulation on the glabrous hand produced the largest spatial extent of the evoked BOLD responses at ‘25 cm/s > No stimulus’, which corresponds closely to the zenith of perceptual capacity for tactile traverse velocity (5 to 30 cm/sec) revealed by human skin psychophysics using a traversing soft brush. Although not a continuous input, the highly effective nature of saltatory pneumotactile inputs on the glabrous hand at 5 cm/s and 25 cm/s shares features of the optimal operating range for discrimination of velocity observed psychophysical studies using continuous brush stimulation applied to the glabrous skin in humans [55]. Parallels in optimal stimulus of perceptual velocity can be drawn from the results of single-unit recordings in non-human primate somatosensory cortex during continuous skin brushing [4, 38, 56].

We have demonstrated regional neural activation using pneumotactile saltatory stimulation via TAC-Cells in both contralateral (20/20 subjects) and ipsilateral somatosensory cortex (6/20 subjects) in in an effort to better understand how various stimulus velocity profiles influence the spatial extent and ROI modulation of brain networks in neurotypical adults. Our results showed that the contralateral BOLD responses were found at sensorimotor cortex (S1, S2, M1, SMA, pre- and postcentral gyrus) across the most subjects (19/20 subjects), whereas the ipsilateral BOLD activations were limited to S1, S2, and deep cerebellum only in 6/20 subjects. The predominantly contralateral BOLD response in the hand representation of the sensorimotor cortex is consistent with human fMRI studies using electrical and laser stimulation [57, 58]. In addition, our finding of a significant ipsilateral BOLD response in the ipsilateral cerebellum is consistent with a previous human PET study using finger movements to create tactile stimulation [59]. Additionally, significant BOLD response in deep cerebellum at 25 cm/sec is consistent with its presumed role in sensory information processing for monitoring and optimizing movement using sensory proprioceptive feedback information [60].

Our results also show that the spatial extent of BOLD responses increased dramatically when stimulus velocity was increased from 5 cm/s to 25 cm/s, and significantly decreased or ‘funneled’ at the highest velocity of 65 cm/s. The %BOLD response change in contralateral S1 (BA3b), showed a robust increase as a function of increasing velocity which is consistent with previous fMRI studies using tactile and non-painful electrical stimulation of the median nerve [42, 61, 62]. The evoked response characteristics associated with moving tactile stimulation suggest that the fast-adapting (FA) mechanoreceptors, which are heavily concentrated in the glabrous hand, are sensitive to tactile stimulation velocity [63]. Further, we discovered the ipsilateral BOLD signal in the IPL at ‘25 cm/s > No stimulus’ contrast. The IPL has been hypothesized to play a role in sensorimotor integration [64, 65].

The TAC-Cells developed in our laboratory are safe, non-invasive, simple, rapidly applicable to the skin, fully compatible with MRI/MEG, produce no stimulus artifact, achieve normal ‘physiologic’ recruitment order of primary mechanosensitive afferents, avoid the potential risks associated with direct-current stimulation methods, and are well tolerated by subjects across the lifespan from infancy through adulthood. Most previous studies were limited to study the median nerve using electrical current stimulation, which noted increasing somatosensory thresholds during the stimulation in healthy human subjects [66]. The TAC-Cells represent a natural mode of tactile stimulation via a small pneumatically charged capsule, which can be placed on virtually any skin surface of the body, including the glabrous hand and face. Our multi-channel pneumotactile stimulus array control system (GALILEO) can be programmed to control pulse duration and rise/fall times, relative timing between individual channels or cells to create unique velocity and direction trajectories over the skin, stimulus block or event-related design (continuous, random, random-balanced), and various triggering modes which are essential for task- or stimulus-related fMRI experiments.

Results of this study have generated new information on the spatiotemporal features of saltatory tactile velocity encoding originating from A*β* mechanoreceptors in the glabrous hand projecting along the medial lemniscus to cerebral and deep cerebellar somatosensory representations in neurotypical adults. Moreover, this work is expected to inform future investigations whose goal is to develop new approaches to motor rehabilitation through somatosensory neurotherapeutics to improve sensorimotor function in individuals who have sustained cerebrovascular stroke or traumatic brain injury. Although the current generation of 3T fMRI scanners provides relatively high spatial resolution (∼2 mm), the temporal resolution is limited (seconds) due to intrinsic properties of the hemodynamic response [67]. The use of a multiband echoplanar sequence can reduce the TR from 2.5 seconds to 1.0 second, thus offering some improvement in BOLD time series modeling. In addition, 7T fMRI scanners could be applied which can produce very high resolution functional data (less than 1mm spatial resolution) [68, 69]. Multimodal combination of fMRI and EEG, or co-registration studies using SQUID-based superconducting MEG, or the rapidly evolving technology known as atomic (AM) or optically-pumped magnetometers (OPM) would potentially yield the best available spatial and temporal resolution to reveal the dynamics of the human somatosensory brain. Moreover, a detailed analysis of the BOLD response time series in other sensorimotor areas (e.g. BA1, BA2, BA4, and cerebellum) could be employed to develop a model of functional brain connectivity as a function of stimulus traverse velocity.

In summary, we found that the TAC-Cell pneumotactile stimulus array delivered at 3 different velocities on the glabrous hand was highly effective at evoking and modulating BOLD responses among 5 ROIs in primary and secondary sensorimotor cortices and deep cerebellum. The strong dependence of %BOLD change found in the present study maps well to known psychophysical and electrophysiological findings in animal and human models and shows potential relevance for motor control of the hand. The spatial extent of BOLD responses was also significantly dependent on the velocity of tactile stimuli.
